# Various Mobile Genetic Elements Involved in the Dissemination of the Phenicol-Oxazolidinone Resistance Gene *optrA* in the Zoonotic Pathogen Streptococcus suis: a Nonignorable Risk to Public Health

**DOI:** 10.1128/spectrum.04875-22

**Published:** 2023-04-18

**Authors:** Xingyang Dai, Junjie Sun, Boqin Zhu, Mingsiyi Lv, Liye Chen, Li Chen, Xiaoming Wang, Jinhu Huang, Liping Wang

**Affiliations:** a MOE Joint International Research Laboratory of Animal Health and Food Safety, College of Veterinary Medicine, Nanjing Agricultural University, Nanjing, China; b Risk Assessment Center of Veterinary Drug Residue and Antimicrobial Resistance, Nanjing Agricultural University, Nanjing, China; c Center for Veterinary Drug Research and Evaluation, Nanjing Agricultural University, Nanjing, China; Universitat Greifswald

**Keywords:** *Streptococcus suis*, PhO resistance, *optrA*, horizontal gene transfer, IS*1216E*, antimicrobial resistance, mobile genetic elements

## Abstract

The rapid increase of phenicol-oxazolidinone (PhO) resistance in Streptococcus suis due to transferable resistance gene *optrA* is a matter of concern. However, genetic mechanisms for the dissemination of the *optrA* gene remain to be discovered. Here, we selected 33 *optrA*-positive S. suis isolates for whole-genome sequencing and analysis. The IS*1216E* element was present in 85% of the *optrA*-carrying contigs despite genetic variation observed in the flanking region. IS*1216E-optrA*-carrying segments could be inserted into larger mobile genetic elements (MGEs), including integrative and conjugative elements, plasmids, prophages, and antibiotic resistance-associated genomic islands. IS*1216E*-mediated circularization occurred to form the IS*1216E*-*optrA*-carrying translocatable units, suggesting a crucial role of IS*1216E* in *optrA* spreading. Three *optrA*-carrying MGEs (ICE*Ssu*AKJ47_*SSU1797*, plasmid pSH0918, and prophage ΦSsuFJSM5_*rum*) were successfully transferred via conjugation at different transfer frequencies. Interestingly, two types of transconjugants were observed due to the multilocus integration of ICE*Ssu*AKJ47 into an alternative *SSU1943* attachment site along with the primary *SSU1797* attachment site (type 1) or into the single *SSU1797* attachment site (type 2). In addition, conjugative transfer of an *optrA*-carrying plasmid and prophage in streptococci was validated for the first time. Considering the abundance of MGEs in S. suis and the mobility of IS*1216E*-*optrA*-carrying translocatable units, attention should be paid to the potential risks to public health from the emergence and spread of PhO-resistant S. suis.

**IMPORTANCE** Antimicrobial resistance to phenicols and oxazolidinones by the dissemination of the *optrA* gene leads to treatment failure in both veterinary and human medicine. However, information about the profile of these MGEs (mobilome) that carry *optrA* and their transferability in streptococci was limited, especially for the zoonotic pathogen S. suis. This study showed that the *optrA*-carrying mobilome in S. suis includes integrative and conjugative elements (ICEs), plasmids, prophages, and antibiotic resistance-associated genomic islands. IS*1216E*-mediated formation of *optrA*-carrying translocatable units played important roles in *optrA* spreading between types of MGEs, and conjugative transfer of various *optrA*-carrying MGEs (ICEs, plasmids, and prophages) further facilitated the transfer of *optrA* across strains, highlighting a nonignorable risk to public health of *optrA* dissemination to other streptococci and even to bacteria of other genera.

## INTRODUCTION

Streptococcus suis is one of the major pathogens of swine, which has been increasingly recognized as an emerging zoonotic agent. Outbreaks of S. suis infections in China and Southeast Asia have caused serious threats to public health (1–3). S. suis is also considered to be an antimicrobial resistance reservoir contributing to the spread of antibiotic resistance genes to major streptococcal pathogens (4, 5). High rates of resistance to commonly used antimicrobials including tetracyclines and macrolides-lincomycins-streptogramin B (MLS_B_) have been reported worldwide in S. suis in the past decades (4, 5). More recently, a rapid increase of S. suis resistant to phenicols and oxazolidinones (PhO) has been documented (6, 7).

Oxazolidinones, including linezolid, tedizolid, and contezolid, represent the last-resort antimicrobial agents against infections caused by multidrug-resistant Gram-positive pathogens (8, 9). However, several studies had confirmed that the exclusive use of florfenicol in veterinary medicine could coselect PhO resistance (10, 11). The PhO resistance in bacteria was originally associated with mutations in the domain V region of 23S rRNA and in genes coding for ribosomal proteins L3, L4, and L22 (12). This situation had changed in 2000, when the first transferable oxazolidinone resistance gene, *cfr*, was identified and reported in a Staphylococcus sciuri isolate (13). Successively, *cfr* variants, *optrA*, and *poxtA* have recently been reported in a variety of Gram-positive and -negative bacteria (14, 15). To date, only *cfr* and *optrA* have been detected in S. suis, with the latter predominantly being isolated (16, 17). For instance, a screening of resistance mechanisms in PhO-resistant S. suis showed that the genes *optrA* and *cfr* were present in 100% and 2.4% of the isolates, respectively (6).

The *optrA* gene encodes an ABC-F superfamily protein which confers PhO resistance by targeting the large subunit of the ribosome. Since the first report of *optrA* in enterococci from humans and food-producing animals (11), the genetic basis responsible for its dissemination has been extensively studied in enterococci, involving different mobile genetic elements (MGEs), particularly the plasmids and transposons (15, 18–20). The *optrA* gene was frequently flanked by a variety of insertion sequences (ISs), including IS*1216E* (21), IS*Efa15* (22), IS*Chh1*-like (23) and IS*Vlu1* (24), which might have facilitated the dissemination of the *optrA* gene across strains, species, or even genus boundaries. However, the transmission of antibiotic resistance genes in streptococci is primarily mediated by integrative and conjugative elements (ICEs) and the recently emerged prophages (5, 25, 26). ICEs are self-transmissible MGEs that primarily reside in the host cell’s chromosome and yet have the ability to be transferred between cells by conjugation (27). ICEs encode the conjugation machinery not only for their self-transfer but also to mobilize other MGEs, including integrative and mobilizable elements and mobilizable genomic islands (28, 29). Until now, information about the profile of these MGEs (mobilome) that carry *optrA* and their transferability in streptococci was limited, especially for the zoonotic pathogen S. suis (7, 26, 30–32).

In this study, we investigated the *optrA*-carrying mobilome in S. suis strains isolated from our previous PhO resistance surveillance project (6, 7) and characterized various *optrA*-carrying MGEs, including ICEs, plasmids, prophages, and antibiotic resistance-associated genomic islands. We further evaluated the role of the IS*1216E* element in dissemination of *optrA* and the transferability of *optrA*-carrying MGEs. Our work demonstrated critical roles of IS*1216E* in mediating movement of *optrA* between types of MGEs. The conjugative transfer of various *optrA*-carrying MGEs (ICEs, plasmids, and prophages) further facilitated the transfer of *optrA* in S. suis.

## RESULTS AND DISCUSSION

### Analysis of *optrA*-carrying mobilome in S. suis.

**FIG 1:**
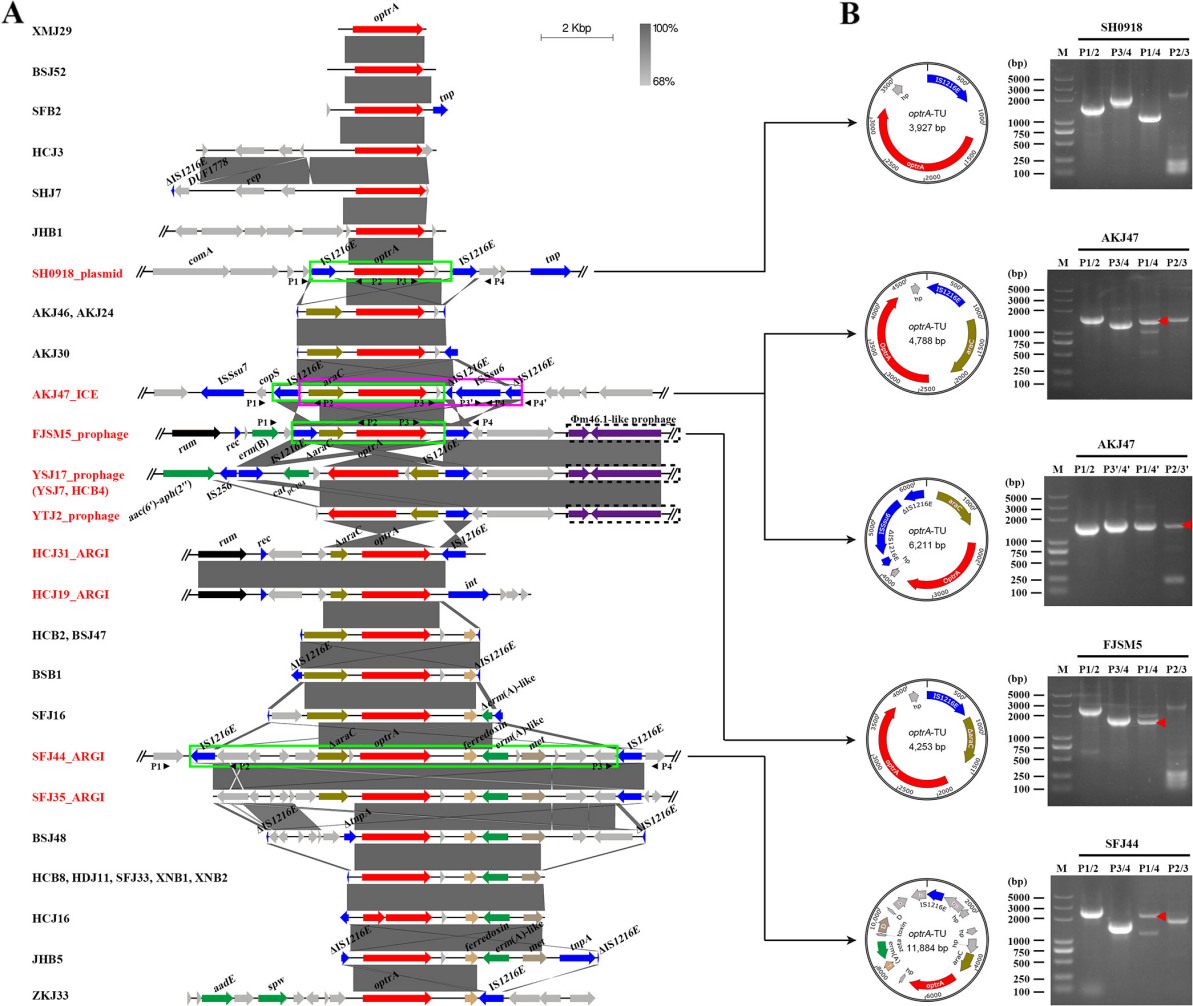
Analysis of *optrA* segment genetic context and detection of IS*1216E*-mediated circular intermediates. (A) Schematic presentation and comparison of the genetic context of *optrA* investigated in this study (*n* = 33). Genes are indicated as arrows, with the arrowhead showing the direction of transcription. The *optrA* gene is marked in red; integrase, transposase, and IS genes are highlighted in blue; other resistance genes are marked in green. Gray shading indicates regions of ≥68% nucleotide sequence identity. Gray triangles indicate primer positions. (B) Schematic presentation of IS*1216E*-mediated circularization of the *optrA* segments in S. suis strains SH0918 (pSH0918), AKJ47 (ICE*Ssu*AKJ47_*SSU1797*), FJSM5 (ΦSsuFJSM5_*rum*), and SFJ44 (ARGI*Ssu*SFJ44_*optrA*). PCR amplification of the presence of both the integrated form (P1/2 and P3/4) and the excised (P1/4)/circular intermediate form (P2/3) of the *optrA* segment. The position of the red triangles corresponds to the expected electrophoretic bands. The products were further sequenced, and the circular form presented at left was confirmed.

We previously observed a rapid increase of *optrA*-mediated PhO resistance among S. suis isolates (6, 7). To further characterize the genetic basis for this rapid dissemination of *optrA* among S. suis isolates, a total of 33 *optrA*-positive S. suis isolates were subjected to whole-genome sequencing (WGS) (see Table S1 in the supplemental material). WGS analysis identified the presence of *optrA*-carrying contigs which ranged from a 2,459-bp single *optrA*-carrying contig to a 12,571-bp segment comprising the IS*1216E*-4*hp*-Δ*araC*-*optrA*-*hp*-*erm*(A)-like-*met*-2*hp*-IS*1216E* structure (Fig. 1A). Most *optrA*-carrying contigs had an *araC*-*optrA* organization (*n* = 16) or an *optrA*-*erm*(A)-like structure (*n* = 11) (Fig. 1A). Despite genetic variation observed in the flanking region of the *optrA* gene, insertion sequence IS*1216E* or truncated IS*1216E* (ΔIS*1216E*) was present upstream or downstream of *optrA* in 26 of the 33 isolates (Fig. 1A and Table S1), suggesting a critical role of IS*1216E* in the spreading of *optrA* among S. suis isolates.

Due to the limitation of sequencing depth, flanking sequences are missing in many *optrA*-carrying contigs. Nevertheless, two copies of IS*1216E* were found in the upstream and downstream regions of *optrA* in the same orientation in sequenced contigs (Fig. 1A), including the smallest IS*1216E*-*optrA-hp*-IS*1216E* (4,608 bp), the IS*1216E*-*araC*-*optrA-hp*-ΔIS*1216E* (6,892 bp), the IS*1216E*-Δ*araC*-*optrA-hp*-ΔIS*1216E* (4,934 bp), and the largest IS*1216E*-4*hp*-Δ*araC*-*optrA*-*hp*-*erm*(A)-like-*met*-2*hp*-IS*1216E* segments, apart from the previously identified IS*1216E*-*araC*-*optrA*-*hp*-*cat*_pC194_-IS*1216E* (6,446 bp) in strain YSJ17 (7). In addition, the *optrA*-carrying segments could be inserted into larger MGEs, including the IS*1216E*-*araC*-*optrA*-*hp*-IS*1216E* integrated into ICEs, prophages, and antibiotic resistance-associated genomic islands from multiple strains, the IS*1216E*-*optrA-hp*-IS*1216E* integrated into plasmid pSH0918 from the early isolate SH0918 in 2009, and the IS*1216E*-4*hp*-Δ*araC*-*optrA*-*hp*-*erm*(A)-like-*met*-2*hp*-IS*1216E* integrated into ARGI1 from strain SFJ44 (Fig. 1A). The integration of *optrA*-carrying segments into different MGEs provide the possibility for their intra- and interspecific transfer by hijacking MGE-encoded transfer machinery.

### IS*1216E* elements mediate the generation of *optrA*-carrying TUs.

Two identical or closely related copies of the IS elements flanking *optrA* might be capable of circularization, thus generating a circular form containing one intact IS element and the sequence between the two copies of IS elements, known as translocatable units (TUs) (33), which can integrate into an ICE or a plasmid or t different chromosomal sites, thereby fostering the dissemination of *optrA* (Fig. 1A). To verify if this was the case in S. suis, the presence of *optrA*-carrying TUs mediated by IS*1216E* was examined by PCR (Fig. 1A). As expected, all the IS*1216E-optrA*-carrying transposons in the strains SH0918, AKJ47, FJSM5, and SFJ44 could be circularized to generate the IS*1216E-optrA*-carrying TUs (Fig. 1B). Interestingly, due to the downstream IS*1216E* being interrupted by IS*Ssu6*, we observed two types of TUs in strain AKJ47, IS*1216E*-*araC*-*optrA*-*hp* (4,788 bp) and *araC*-*optrA*-*hp-*ΔIS*1216E-*IS*Ssu6-*ΔIS*1216E* (6,211 bp), respectively, indicating that the IS*1216E* element from either upstream or downstream of *optrA* could drive the formation of TUs. These results further suggested that IS*1216E*-mediated circularization of IS*1216E-optrA*-carrying transposons from different MGEs might have played an important role in facilitating its dissemination between different MGEs, bacterial strains, and even species (15, 21).

### Conjugative transfer of *optrA* between S. suis strains.

To test transferability of the *optrA* gene located on different MGEs, conjugation experiments were performed using the above-mentioned four strains with different *optrA*-carrying MGEs as donors. Transfer was observed in strains AKJ47, SH0918, and FJSM5 but not in SFJ44 (Table 1). The three corresponding transconjugants cAKJ47, cSH0918, and cFJSM5 exhibited elevated MICs to linezolid (4 to 8 mg/L) and florfenicol (32 to 64 mg/L) (Table 1). In addition, transconjugant cFJSM5 also exhibited resistance to erythromycin (MIC, 32 mg/L) and clindamycin (MIC, 64 mg/L) (Table 1), suggesting the cotransfer of the upstream *erm*(B) with the IS*1216E*-Δ*araC*-*optrA-hp*-ΔIS*1216E* transposon. The donor strains and their corresponding transconjugants cAKJ47, cSH0918, and cFJSM5 were further completely genomically sequenced for the characterization of the MGEs responsible for *optrA* transfer, confirming that interstrain transfer of *optrA* was mediated by the self-transferable MGEs (ICE*Ssu*AKJ47_*SSU1797*, plasmid pSH0918, and prophage ΦSsuFJSM5_*rum*; see below) rather than the IS*1216E-optrA*-carrying TUs, under laboratory experimental conditions.

**TABLE 1:** Characteristics of the donors and their transconjugants in transfer experiments^*c*^

Strain^*a*^	Serotype	MLST	Transfer frequency^*b*^	Transferred MGE [resistance gene(s)]	MIC (mg/L)				
					LZD	FFC	ERY	CLI	TET
P1/7RF	2	1			<0.5	0.5	<0.5	<0.5	0.5
SH0918	5	499		pSH0918 [*optrA*]	8	32	>256	128	16
cSH0918			(5.70 ± 1.06) × 10^−8^		8	32	<0.5	<0.5	<0.5
AKJ47	3	662		ICE*Ssu*AKJ47_*SSU1797* [*optrA*]	4	32	>256	256	32
cAKJ47			(5.99 ± 1.53) × 10^−6^		8	32	<0.5	<0.5	<0.5
FJSM5	31	1593		ΦSsuFJSM5_*rum* [*optrA*, *erm*(B)]	4	64	>256	>256	16
cFJSM5			(1.01 ± 2.71) × 10^−8^		4	32	>256	64	<0.5
SFJ44	NT	1087			4	16	>256	>256	128
cSFJ44			NS		−	−	−	−	−

a S. suis SH0918, AKJ47, FJSM5, and SFJ44 were used as donors while S. suis P1/7RF was used as recipient isolate, and cSH0918, cAKJ47, cFJSM5, and cSFJ44 were transconjugants.

b Transfer frequency was calculated by CFU of transconjugants per donor.

c LZD, linezolid; FFC, florfenicol; ERY, erythromycin; CLI, clindamycin; TET, tetracycline; NT, nontypeable; − or NS, not successful; MLST, multilocus sequence type. MICs shaded gray represent strains that were resistant to the corresponding antimicrobial agents.

Differences in transfer frequencies were observed between ICEs, plasmids, and prophages. The ICE*Ssu*AKJ47_*SSU1797* exhibited much higher transfer frequency than pSH0918 (~100-fold) and ΦSsuFJSM5_*rum* (~500-fold) (Table 1). One possible explanation is that the ICE*Ssu*AKJ47_*SSU1797* encoded the intact conjugation machinery for its self-transfer at a much higher transfer frequency, while the plasmid pSH0918 and prophage ΦSsuFJSM5_*rum* lacked conjugation machinery. However, a coresident ICE was present on both host chromosomes (data not shown), suggesting that the successful transfer of pSH0918 and ΦSsuFJSM5_*rum* might be mobilized by ICE at low frequency. It is reasonable that ICEs were distributed more frequently than plasmids and prophages in streptococci (5, 25, 26).

### Transfer and multilocus integration of a novel *optrA*-carrying ICE, ICE*Ssu*AKJ47_*SSU1797*.

**FIG 2:**
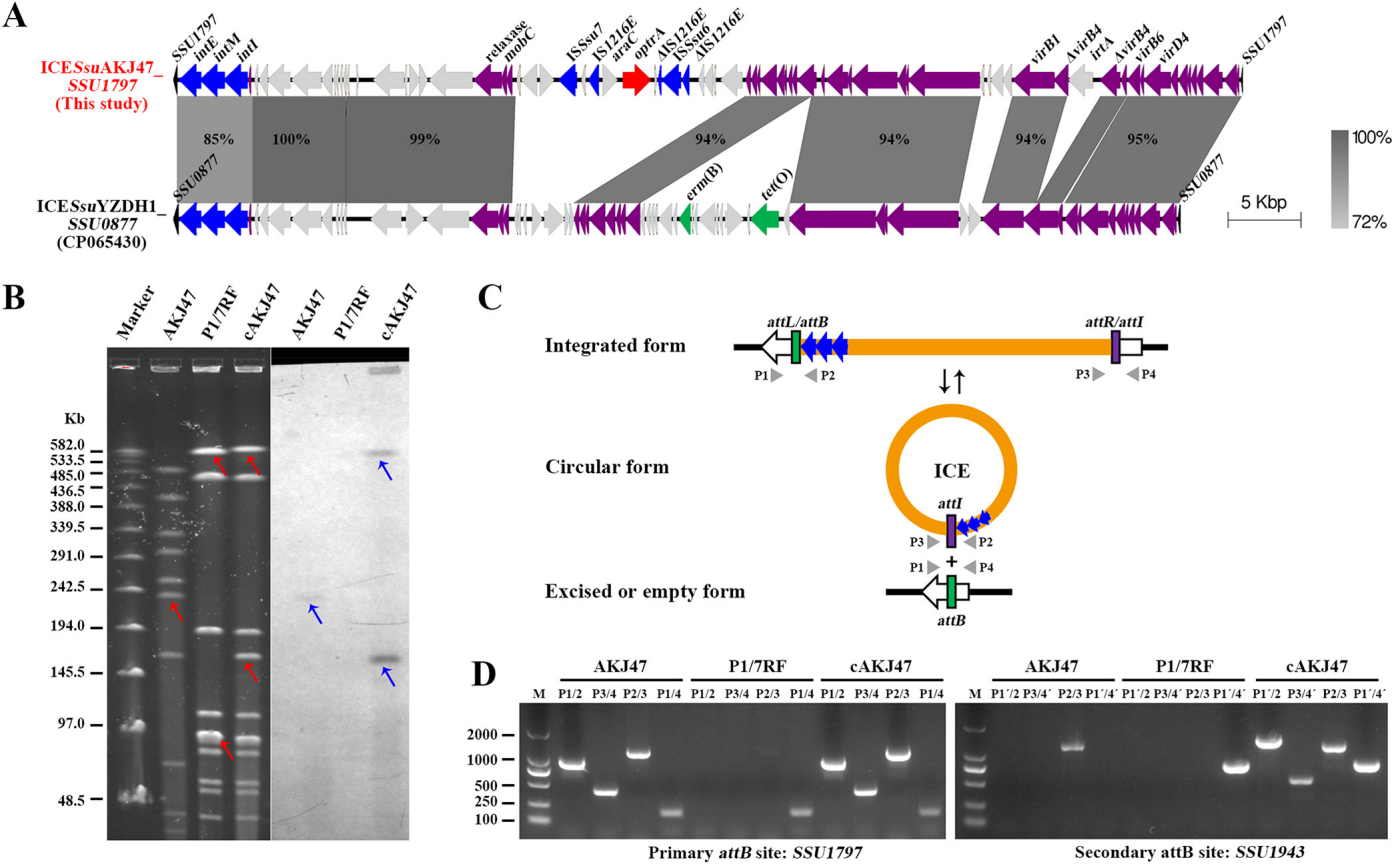
Characterization of *optrA*-carrying ICE*Ssu*AKJ47_*SSU1797* in S. suis AKJ47. (A) Comparison of the ICE*Ssu*AKJ47_*SSU1797* carrying *optrA* identified in this study with ICE*Ssu*YZDH1 in S. suis. Bacterial chromosomal genes are shown in black, ICE backbone genes are shown in purple, and variable genes are shown in light gray. Integrase and IS genes are highlighted in blue, *optrA* gene is marked in red, and other resistance genes are marked in green. Gray shading indicates regions of ≥72% nucleotide sequence identity. (B) SmaI-PFGE fingerprint patterns (left panel) and Southern blot hybridization with *optrA* probe (right panel) of the S. suis AKJ47 conjugation pairs. Marker, the standard molecular mass of Lambda PFG ladder. The transfer of *optrA*-carrying ICE is inferred by red arrows. The location of probed *optrA* is highlighted by blue arrows. (C) Schematic representation of integration and excision/circularization states of ICE*Ssu*AKJ47_*SSU1797* and location of PCR primers used to detect excision and circular form. (D) PCR amplification of the presence of both integrated form (*attL* and *attR*) and excised/episome form (*attB* and *attI*) of ICE*Ssu*AKJ47_*SSU1797* in donor AKJ47, recipient P1/7RF, and transconjugant cAKJ47. Primer pairs P1/2, P3/4, P1/4, and P2/3 were used to detect *attL*, *attR*, *attB*, and *attI* sites targeting *SSU1797* or *SSU1943*, respectively.

In strain AKJ47, the *optrA*-carrying ICE*Ssu*AKJ47_*SSU1797* was 68,648 bp in length and comprised 89 predicted open reading frames (ORFs) with an imperfect direct repeat of 2 or 4 nucleotides (nt) (5′-GC-3′/5′-TCCC-3′) in its flanking region, which was integrated into the *SSU1797* gene, encoding a MutT/Nudix family protein, MutX (Fig. 2A). The ICE*Ssu*AKJ47_*SSU1797* backbones (except the integration module) exhibited 94.1 to 99.3% identity to ICE*Ssu*YZDH1 at the *SSU0877* gene (encoding another MutT/Nudix family hydrolase) in S. suis strain YZDH1, the founder of a widespread family of ICEs in S. suis (34), the integration modules of which were composed of three serine integrases in triplets, external (E), middle (M), or internal (I), and allowed their integration into one to three of the four integration sites (*SSU0468*, *SSU0877*, *SSU1262*, and *SSU1797*) in the host genome (34). Unlike the previously reported ICE*Ssu*YZDH1 family of ICEs, which contributed to the global dissemination of *tet*(O)- and *erm*(B)-mediated tetracyclines and MLS_B_ resistance, the novel ICE*Ssu*AKJ47_*SSU1797* lacked *tet*(O) and *erm*(B) genes but carried the IS*1216E*-*araC*-*optrA*-*hp-*ΔIS*1216E-*IS*Ssu6-*ΔIS*1216E* within hot spot HS-2 (25), warning of the potential transmission of *optrA*-mediated PhO resistance in S. suis by the ICE*Ssu*YZDH1 family of ICEs.

The transfer frequency of ICE*Ssu*AKJ47_*SSU1797* to the recipient strain S. suis P1/7RF was (5.99 ± 1.53) × 10^−6^, a rate similar to that of ICE*Ssu*YZDH1_*SSU0877* and higher than that of the ICE*Sa*2603 family of ICEs (34, 35). DNA hybridization of AKJ47 conjugation pairs with a specific probe for *optrA* suggested that the transferred ICE*Ssu*AKJ47_*SSU1797* was located on an ~160-kb band and an ~590-kb band, respectively (Fig. 2B), indicating that the acquisition of ICE occurred by stable integration into additional attachment sites (secondary integration site) except for the primary *SSU1797* site. Complete genome analysis of transconjugant cAKJ47 revealed the presence of an additional copy of ICE*Ssu*AKJ47 by integration into the *SSU1943* gene (encoding another MutT/Nudix family hydrolase), a previously nonreported integration site. Sequence analysis revealed the presence of imperfect direct repeats flanking the ICE at the *SSU1943* site (5′-AC-3′/5′-TCCC-3′). One hundred positive clones in transconjugants from conjugation experiments were then randomly selected and screened for the multilocus integration of ICE*Ssu*AKJ47. Subsequently, two types of transconjugants were identified, one with ICE*Ssu*AKJ47 integrated at both *SSU1797* and *SSU1943* sites (type 1), and the other integrated at only the *SSU1797* site (type 2), accounting for 82.0% and 18.0% of the transconjugants, respectively. To test the ability of ICE*Ssu*AKJ47 to integrate into and excise from the primary site (*SSU1797*) and the secondary site (*SSU1943*), an inverse PCR amplification followed by Sanger sequencing analysis was performed (Fig. 2C). PCR amplification confirmed the integration and excision/circularization of ICE*Ssu*AKJ47 in both sites in transconjugant cAKJ47, but for donor strain AKJ47, only one copy of ICE*Ssu*AKJ47 was observed at the primary *SSU1797* site (Fig. 2D). We did not observe the integration of ICE*Ssu*AKJ47_*SSU1797* into *SSU0468*, *SSU0877*, and *SSU1262* sites in donors and transconjugants (data not shown). Accumulation of IS*1216E-optrA*-carrying TUs into the novel ICE*Ssu*YZDH1 family of ICEs with its multilocus integration into different attachment sites of the host bacteria could have benefited from its dissemination, posing a great challenge to the control of *optrA* spreading.

### Characterization and transfer of a novel *optrA*-carrying plasmid, pSH0918.

**FIG 3:**
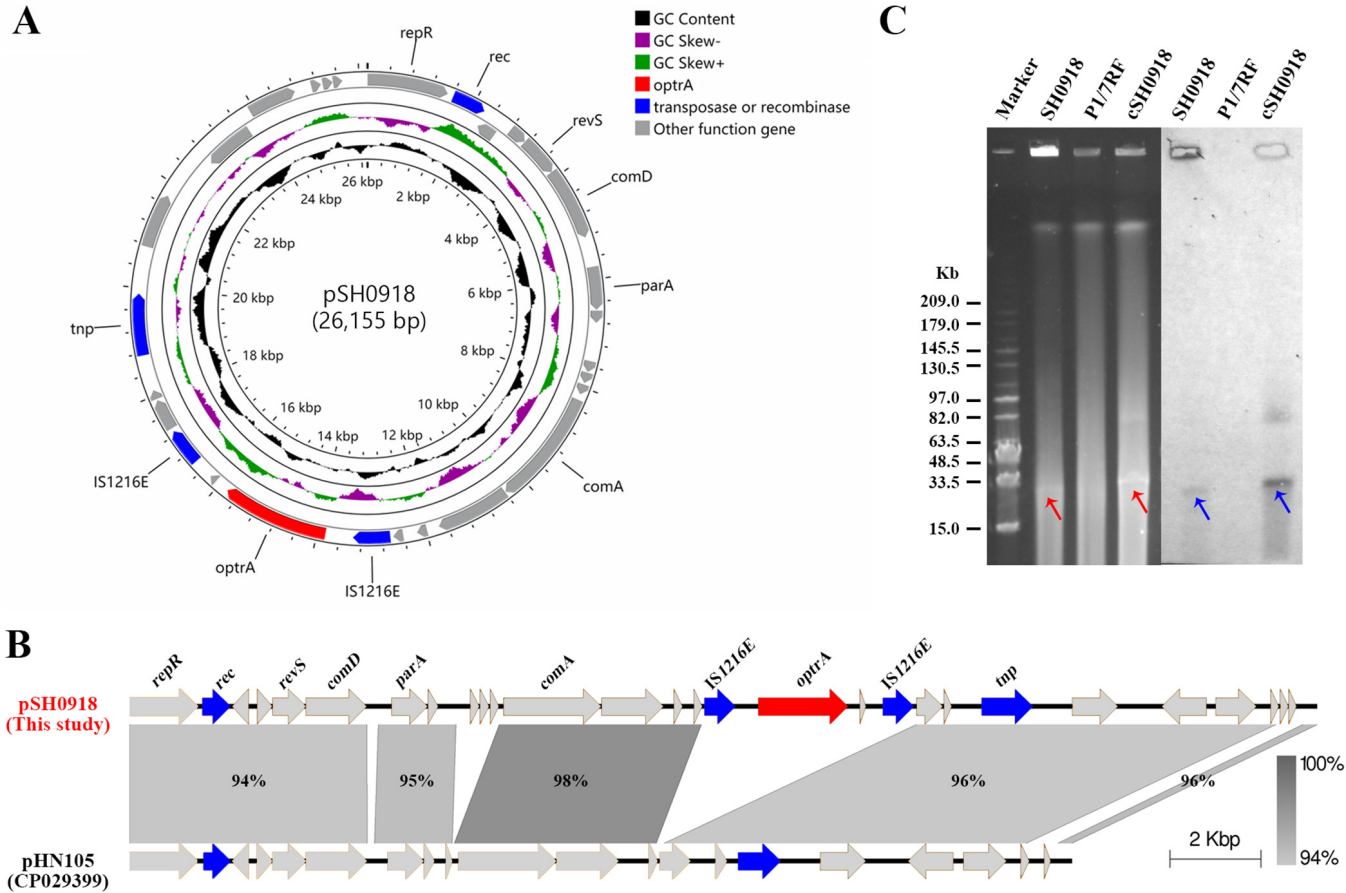
Characterization of *optrA*-carrying plasmid pSH0918 in S. suis SH0918. (A) Circular genome maps of plasmid pSH0918 from outside to inside indicate the following: predicted coding sequences, GC skew, GC content, and scale in kilobases. (B) Linear comparison of the plasmid pSH0918 identified in this study with plasmid pHN105 in S. suis. Gray shading indicates regions of ≥94% nucleotide sequence identity. (C) S1-PFGE fingerprint patterns (left panel) and Southern blot hybridization with *optrA* probe (right panel) of the S. suis SH0918 conjugation pairs, respectively. Marker, the standard molecular mass of MidRange PFG marker. The transfer of *optrA*-carrying plasmid is inferred by red arrows. The location of probed *optrA* is highlighted by blue arrows. In panels A and B, the *optrA* gene is marked in red, recombinase, transposase, and IS genes are highlighted in blue, and other genes are shown in light gray.

The *optrA*-carrying plasmid pSH0918 in strain SH0918 was 26,155 bp in length and comprised 28 predicted ORFs (Fig. 3A), with an average lower GC content (33.2%) than the chromosome (41.1%). The *repR* gene of plasmid pSH0918 had 95.6% identity to the replicon of the broad-host-range Inc18 family plasmids, which have been associated with the dissemination of a variety of resistance genes, including the MLS_B_ resistance gene *erm*(B), the tetracycline resistance gene *tet*(S), the vancomycin resistance gene *vanA*, and, more recently, the oxazolidinone resistance genes *optrA* and *poxtA* among Gram-positive coccus isolates (36, 37). Sequence analysis showed that the nonconjugative plasmid pSH0918 shared conserved core structure with plasmid pHN105 (38). The insertion of the 4.7-kb transposon IS*1216E*-*optrA-hp-*IS*1216E* into the core promoter-binding protein (CPBP) family intramembrane metalloprotease gene at the 5′ start position might have resulted in the acquisition of *optrA* by plasmid pSH0918 (Fig. 3B). This was supported by the observation of integration and excision of IS*1216E*-*optrA-hp-*IS*1216E* from the plasmid (Fig. 1B).

Previous reports have shown that the *optrA* gene can be carried by plasmids of enterococci and staphylococci, while for S. suis, a single report of plasmid-borne *optrA* was recently described (30). However, the *optrA*-carrying locus on the plasmid of HNAY3 had a single IS*Enfa1*-like element upstream of *optrA*, and this plasmid was transferred into Staphylococcus aureus and S. suis by electrotransformation rather than conjugation (30), which prompted us to continue exploring the role of IS elements in transmission of *optrA* into plasmids and the transferability of *optrA*-carrying plasmids between strains. For the first time, we demonstrated the conjugative transfer of the *optrA*-carrying plasmid pSH0918 to the recipient strain S. suis P1/7RF at a frequency of (5.70 ± 1.06) × 10^−8^. S1-pulsed-field gel electrophoresis (PFGE) and DNA hybridization revealed that *optrA* was transferred along with the plasmid pSH0918 (Fig. 3C).

### Identification and transfer of a novel *optrA*-carrying prophage, ΦSsuFJSM5_*rum*.

**FIG 4:**
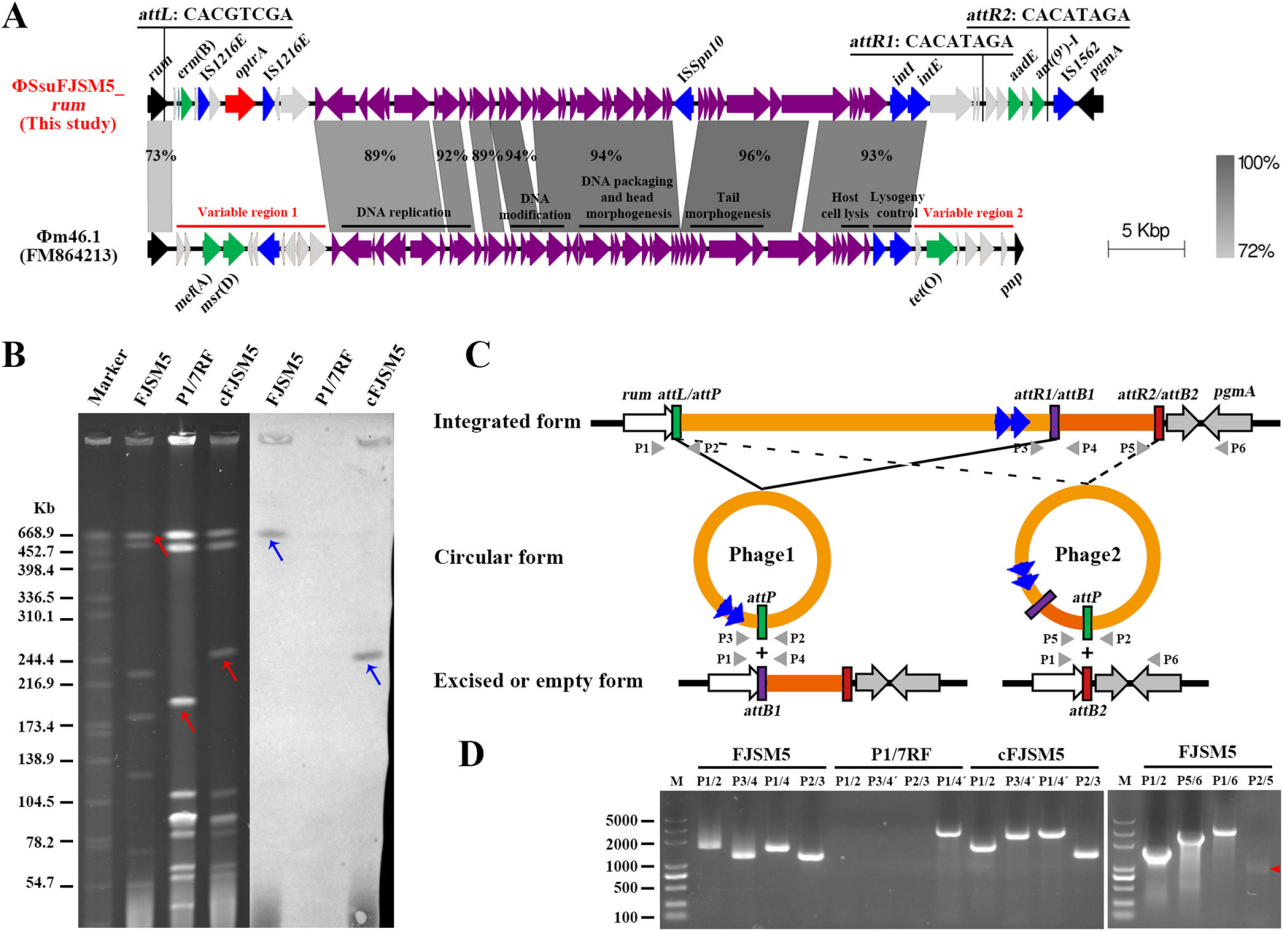
Characterization of *optrA*-carrying prophage ΦSsuFJSM5_*rum* in S. suis FJSM5. (A) Comparison of the prophage ΦSsuFJSM5_*rum* carrying *optrA* identified in this study with Φm46.1 in S. pyogenes. Bacterial chromosomal genes are shown in black, prophage backbone genes are shown in purple, and variable genes are shown in light gray. Integrase and IS genes are highlighted in blue, the *optrA* gene is marked in red, and other resistance genes are marked in green. Gray shading indicates regions of ≥72% nucleotide sequence identity. (B) SmaI-PFGE fingerprint patterns (left panel) and Southern blot hybridization with *optrA* probe (right panel) of the S. suis FJSM5 conjugation pairs, respectively. Marker, the standard molecular mass of Salmonella enterica serovar Braenderup H9812. The transfer of *optrA*-carrying prophage is inferred by red arrows. The location of probed *optrA* is highlighted in blue arrows. (C) Schematic representation of integration and excision/circularization states of small and larger prophage ΦSsuFJSM5_*rum* and location of PCR primers used to detect excision and circular form. (D) PCR amplification of the presence of both integrated form (*attL* and *attR*) and excised/episome form (*attB* and *attP*) of prophages in donor FJSM5, recipient P1/7RF, and transconjugant cFJSM5. Primer pairs P1/2, P3/4 (or P5/6 and P3/4′), P1/4 (or P1/6 and P1/4′), and P2/3 (or P2/5) were used to detect *attL*, *attR*, *attB*, and *attP* sites, respectively.

The *optrA* gene in strain FJSM5 was located on a prophage at the *rum* site (Fig. 4A). The ΦSsuFJSM5_*rum* was 57,765 bp in length and comprised 64 predicted ORFs. ΦSsuFJSM5_*rum* shared conservative syntenic core structure with Streptococcus pyogenes prophage Φm46.1 (39), which contains a conserved region with six modules flanked by two variable regions (VRs) (Fig. 4A). The *optrA* gene and the MLS_B_ resistance gene *erm*(B) were located on VR1, while the aminoglycoside resistance genes *aadE* and *ant(9*′)-*I* accumulated on VR2. Interestingly, Φm46.1 and related prophages have been documented in many streptococcal species, which carried determinants of resistance to MLS_B_, *mef*(A), *erm*(B), *lnu*(B), and *lnu*(C); tetracyclines, *tet*(O), *tet*(O/W/32/O), and *tet*(W); aminoglycosides, *ant(6)*-*Ib*, *aphA3*, *sat4*, and *aadE*; and PhO, *cat*_PC194_ and *optrA* (40), highlighting that antibiotic resistance genes can also be transferred by prophages. Currently, two *optrA*-carrying prophages have been reported, including ΦSC181, which also carried *mef*(A), *aacA-aphD*, and *cat*, and ΦSsuYSJ17-3, which also contained *erm*(B), *aphA3*, *aac(6′)-aph(2′′)*, and *cat* (7, 17, 26); however, transfer of these prophages was not successful (7, 26).

Transfer of ΦSsuFJSM5_*rum* using a standard transduction protocol was not achievable even after more than three attempts. We noted *in vitro* evidence of transfer of prophage Φm46.1. However, the transfer experiment was performed with a virtual conjugation protocol (41), suggesting the transfer of prophage was assisted by conjugative elements via conjugation. To test this possibility, conjugative transfer of ΦSsuFJSM5_*rum* was performed. FJSM5 transferred the *optrA* gene to the recipient strain S. suis P1/7RF by conjugation at a frequency of (1.01 ± 2.71) × 10^−8^. SmaI-PFGE and DNA hybridization revealed that *optrA* was transferred along with the self-transfer prophage ΦSsuFJSM5_*rum* (Fig. 4B). Complete genome analysis of transconjugant cFJSM5 showed that the resistance genes *aadE* and *ant(9*′)-*I* were not transferred to the recipient strain with the prophage ΦSsuFJSM5_*rum*. Further analysis showed that the ΦSsuFJSM5_*rum* region was bounded by a single *attL* and two *attR* sites (*attR1* and *attR2*); this suggests that two circular episomes may be formed, either a small prophage, ΦSsuFJSM5_*rum*1 (*attL*×*attR1*), or a larger prophage, ΦSsuFJSM5_*rum*2 (*attL*×*attR2*). To verify if this was the case, two circular forms of prophage were tested by inverse PCR and sequence analysis (Fig. 4C). As expected, two types of prophages could be circularized to form the episome by using P2/P3 (or P2/P5) primer pairs and the empty site *attB* by using P1/P4 (or P1/P6) primer pairs (Fig. 4D). Analysis of the *att* sequences allowed us to identify an imperfect 8-bp nucleotide match (*attL*/*attP*, 5′-CACGTCGA-3′; *attR1*/*attB1*, 5′-CACATAGA-3′; *attR2*/*attB2*, 5′-CACATAGA-3′), which is inconsistent with the 2-bp GA sequence in Φm46.1 of a previous analysis (39). In addition, in the conjugation pairs of FJSM5 and P1/7RF, only the transfer of the small prophage ΦSsuFJSM5_*rum*1 to P1/7RF was observed, which caused the recipient to acquire only the resistance genes *optrA* and *erm*(B).

In conclusion, different *optrA*-carrying MGEs, including plasmids, ICEs, and prophages, and antibiotic resistance-associated genomic islands are associated with the rapid increase of PhO resistance in S. suis. The high prevalence of IS*1216E* flanking the *optrA* gene and IS*1216E*-mediated formation of different IS*1216E-optrA*-carrying TUs might have played a vital role in the dissemination of *optrA* between MGEs. Moreover, the horizontal transfer of *optrA*-carrying ICEs, plasmids, and even prophages might explain the higher prevalence of *optrA* and diversity of MGEs in S. suis than in other streptococci. Considering the abundance of MGEs in S. suis and the mobility of *optrA*-carrying TUs, attention should be paid to the spread of *optrA*-mediated PhO resistance in S. suis, highlighting the risks of horizontal gene transfer (HGT) of *optrA*-carrying MGEs to other streptococci and even to bacteria of other genera.

## MATERIALS AND METHODS

### Bacterial strains and antimicrobial susceptibility testing.

S. suis strains were grown in Todd-Hewitt broth (THB) or on Todd-Hewitt agar (THA) plates supplemented with 5% (vol/vol) calf serum and incubated at 37°C. Antimicrobial susceptibility testing was performed using the broth microdilution method according to CLSI guideline M100-ED29 (42). The antimicrobial agents used in this study are listed in Table 1.

### WGS and analysis.

Genomic DNAs of the isolates were purified and sequenced using the Illumina HiSeq 2000 platform (Novogene, China) and assembled with SOAP *de novo* v.2.04 (43). Gaps between *optrA*-carrying MGEs were closed by PCR with Sanger sequencing. The complete genome of strains in transfer assays was further sequenced using the PacBio RSII system (Novogene, China). Preliminary assembly was conducted with SMRT Link v.5.0.1 and then corrected with whole-genome sequencing (WGS) data. MGEs were identified by ICEfinder (https://bioinfo-mml.sjtu.edu.cn/ICEfinder/index.php), PHASTER (http://phaster.ca/), and ISfinder (https://www-is.biotoul.fr/). Genetic environment analysis of the *optrA*-carrying elements was visualized with Easyfig v.2.2.2 (44).

### PCR amplification analysis.

IS*1216E*-mediated circularization of the *optrA*-carrying TUs was detected by an inverse PCR in selected strains with different genetic organizations, including within the SH0918 plasmid pSH0918, the AKJ47 ICE*Ssu*AKJ47_*SSU1797*, the FJSM5 prophage ΦSsuFJSM5_*rum*, and the SFJ44 ARGI1, followed by sequencing analysis. Integrase-mediated excision and integration of ICE*Ssu*AKJ47_*SSU1797* and prophage ΦSsuFJSM5_*rum* were performed with inverse PCR to detect the extrachromosomal circular form and integrated form of MGEs in both donors and their corresponding transconjugants. The specific primers are listed in Table S2 in the supplemental material.

### Transfer experiments.

The transferability of different *optrA*-carrying MGEs was determined by filter mating assays as described previously (35). Strains SH0918 (with *optrA*-carrying plasmid pSH0918), AKJ47 (with *optrA*-carrying ICE*Ssu*AKJ47_*SSU1797*), FJSM5 (with *optrA*-carrying prophage ΦSsuFJSM5_*rum*), and SFJ44 (with *optrA*-carrying ARGI1) were selected as donors, and S. suis P1/7RF served as a recipient. The transfer frequency was calculated as the mean number of transconjugants per donor in each assay of three duplicates.

### PFGE and DNA hybridization.

The genomic location of *optrA* and the sizes of *optrA*-carrying MGEs in both the donors and related transconjugants were examined by S1-PFGE (for plasmid-borne *optrA*) or SmaI-PFGE (for chromosome-borne *optrA*) as previously described (37, 45), followed by Southern blotting and DNA hybridization using a digoxigenin (DIG)-labeled probe specifically targeting the *optrA* gene with primers listed in Table S2.

### Data availability.

The WGSs of S. suis strains have been submitted to GenBank with the *optrA*-carrying contigs or complete genomes under the following accession numbers (also listed in Table S1): strain SH0918, JAIMEB010000002; strain AKJ24, JAGFQV010000064; strain AKJ30, JANFLY000000061; strain AKJ46, JANFLZ000000064; strain AKJ47, JANFMA000000043; strain BSB1, JANFMB000000060; strain BSJ47, JANFMC000000032; strain BSJ48, JANFMD000000029; strain BSJ52, JANFME000000043; strain HCB2, JANFMF000000061; strain HCB4, QXEP01000058; strain HCB8, JAGFQW010000051; strain HCJ16, JANFMG000000063; strain HCJ19, JANFMH000000006; strain HCJ3, JAFFHR010000042; strain HCJ31, JAIMEP010000008; strain JHB1, JANFMJ000000041; strain JHB5, JANFMK000000056; strain XNB1, JANFMO000000062, strain XNB2, JANFMP000000063; strain YSJ17, CP032064; strain YSJ7, QXEQ01000061; strain YTJ2, JAIMDU010000024; strain ZKJ33, JAFFHS010000028; strain HDJ11, JANFMI000000066; strain SFB2, JANFML000000054; strain SFJ16, JAGFUZ010000030; strain SFJ33, JANFMM000000068; strain SFJ35, JANFMN000000017; strain SFJ44, CP031970; strain SHJ7, JAIMZF010000036; strain XMJ29, JAIMZE010000052; strain FJSM5, CP082204. The complete genomes of plasmid pSH0918 and transconjugants cAKJ47 and cFJSM5 have been submitted to GenBank under accession numbers MT180124, CP102746, and CP082199, respectively.
